# Balancing Effective Mass and Carrier Mobility via Ga‐Induced Multi‐Band Valley Engineering in GeTe Thermoelectrics

**DOI:** 10.1002/advs.74427

**Published:** 2026-02-15

**Authors:** Jianglong Zhu, Yan Zhong, Xiang An, Huangshui Ma, Jie Zheng, Huagang Xiao, Shiyuan Zhan, Pingan Song, Min Hong, Ran Ang

**Affiliations:** ^1^ College of Physics Chengdu University of Technology Chengdu China; ^2^ School of Intelligent Manufacturing Sichuan University of Arts and Science Dazhou China; ^3^ Key Laboratory of Radiation Physics and Technology Ministry of Education Institute of Nuclear Science and Technology Sichuan University Chengdu China; ^4^ Centre for Future Materials School of Science Engineering and Digital Technologies University of Southern Queensland Springfield Campus Queensland Australia; ^5^ College of Energy Chengdu University of Technology Chengdu China

**Keywords:** band engineering, chemical bonding modulation, GeTe‐based alloys, power conversion efficiency, thermoelectrics

## Abstract

Multi‐band valley engineering offers an effective route to achieving high thermoelectric performance; however, the associated increase in the density‐of‐states effective mass (*m* ^*^) inevitably compromises carrier mobility (*µ*), posing a fundamental challenge for further enhancement of the figure of merit (*ZT*). Here, Ga doping is employed to tailor the electronic band structure in Ge_0.94_Bi_0.06_Te, inducing the simultaneous convergence of three valence band edges and the emergence of a midgap band, which markedly enhances *m* ^*^ and the Seebeck coefficient. Reduced electron localization arising from Ga‐Te bonding, together with interfaces featuring small lattice mismatch, effectively mitigates carrier scattering and preserves a high *µ*. As a result, an optimized balance between *m* ^*^ and *µ* yields an outstanding weighted mobility and power factor. Furthermore, Ga‐induced lattice vibration disorder, in synergy with engineered multi‐scale crystal defects, strongly suppresses the lattice thermal conductivity. Consequently, a high *ZT* exceeding 2.1 is achieved at 653 K. A single‐stage lead‐free device based on the optimized material delivers a competitive power conversion efficiency of 7.7% under a temperature difference of 440 K. This study provides new insights into the rational design of high‐performance lead‐free thermoelectric materials and devices.

## Graphical abstract

Ga doping drives simultaneous convergence of three valence bands and formation of a midgap band in Ge_0.94_Bi_0.06_Te, enhancing the Seebeck coefficient without sacrificing carrier mobility. Lattice vibration disorder and multi‐scale defect engineering suppress lattice thermal conductivity, delivering a ZT exceeding 2.1 and enabling efficient lead‐free thermoelectric power conversion devices under large temperature gradients relevant to practical energy applications.

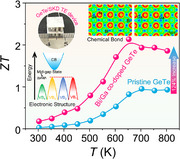

## 1 Introduction

Thermoelectric (TE) technology offers a sustainable route toward carbon neutrality and global energy management by enabling emission‐free conversion of ubiquitous waste heat into electricity [1, 2]. The practical viability of TE devices depends heavily on their energy conversion efficiency (*η*), which is governed by the figure of merit (*ZT*) of TE materials. This metric is expressed as *ZT* = *S* ^2^ *σT*/*κ*, comprising the Seebeck coefficient (*S*), electrical conductivity (*σ*), thermal conductivity (*κ*), and absolute temperature (*T*) [3, 4, 5, 6]. A primary strategy to elevate *ZT* involves decoupling electron and phonon transport: maximizing the power factor (*PF* = *S* ^2^ *σ*) while simultaneously minimizing lattice thermal conductivity (*κ*_lat_ = *κ*‐*κ*_ele_, where *κ*_ele_ denotes the electronic thermal conductivity) [7, 8]. However, the intrinsic coupling of these parameters viathe carrier concentration (*n*_H_) creates a bottleneck that complicates independent optimization. To circumvent this limitation, researchers have adopted multi‐dimensional strategies, such as *n*_H_ tuning, band structure engineering, and nanostructure and defect engineering [9, 10, 11, 12, 13, 14].

Germanium Telluride (GeTe), a narrow‐bandgap (*E*_g_∼0.2 eV) degenerate semiconductor from the IV‐VI group, is widely regarded as a promising *p*‐type TE material for intermediate temperature ranges, distinguished by its combination of high TE performance and mechanical robustness [15, 16]. Pristine GeTe crystallizes in a rhombohedral (*r*‐) structure (*R*3*m*, ferroelectric phase) at room temperature but transforms into a cubic (*c*‐) phase (*Fm* $\bar{3}$ *m*, paraelectric phase) around 700 K via reversible Peierls distortion along the [111] direction [17, 18]. However, this structural instability, arising from the pronounced size mismatch between cations and anions, promotes the formation of intrinsic Ge vacancies under thermodynamic equilibrium. These vacancies act as acceptor defects, yielding an excessive hole carrier density (*n*_H_∼1 × 10^21^ cm^−3^) that degrades TE efficiency. Consequently, chemical doping with species like Pb, Sb, or Bi has become a standard approach to optimize *n*_H_, achieved either by suppressing vacancy formation energies or through aliovalent hole compensation [19, 20, 21].

Nevertheless, strategies restricted solely to *n*_H_ optimization eventually hit an intrinsic ceiling, limiting further gains in *ZT*. In this context, electronic band modulation and phonon transport regulation have emerged as effective strategies for overcoming performance bottlenecks [22, 23, 24]. *r*‐GeTe is an indirect‐bandgap semiconductor, with the valence band maximum (VB) located at the *∑* point and the conduction band minimum (CB) at the *L* point of the Brillouin zone, accompanied by multiple sub‐valence band extrema. Alloying or doping with elements such as Mn [25], Cd [26, 27], Cr [28], Sn [29], Hg [18, 30], Sb [31], and rare‐earth elements [32, 33, 34] reduces the contribution of Ge 4*s* orbitals to the VB edge, thereby minimizing the energy offset (Δ*E*) between the heavy and light valence bands and enabling band convergence, which provides additional transport channels for charge carriers. Moreover, the interaction between transition‐metal *d* orbitals and Te *p* orbitals induces *p‐d* hybridization, modifying the electronic structure and introducing localized impurity states near the band edges, which can effectively enhance *S* [35, 36, 37, 38]. On the phonon side, the introduction of point defects, grain or phase boundaries, nanoscale precipitates, as well as hierarchical nanostructures into the GeTe matrix significantly increases lattice disorder across multiple length scales, thereby intensifying phonon scattering and playing a pivotal role in suppressing *κ*_lat_ [39, 40, 41, 42, 43, 44, 45].

**FIGURE 1:**
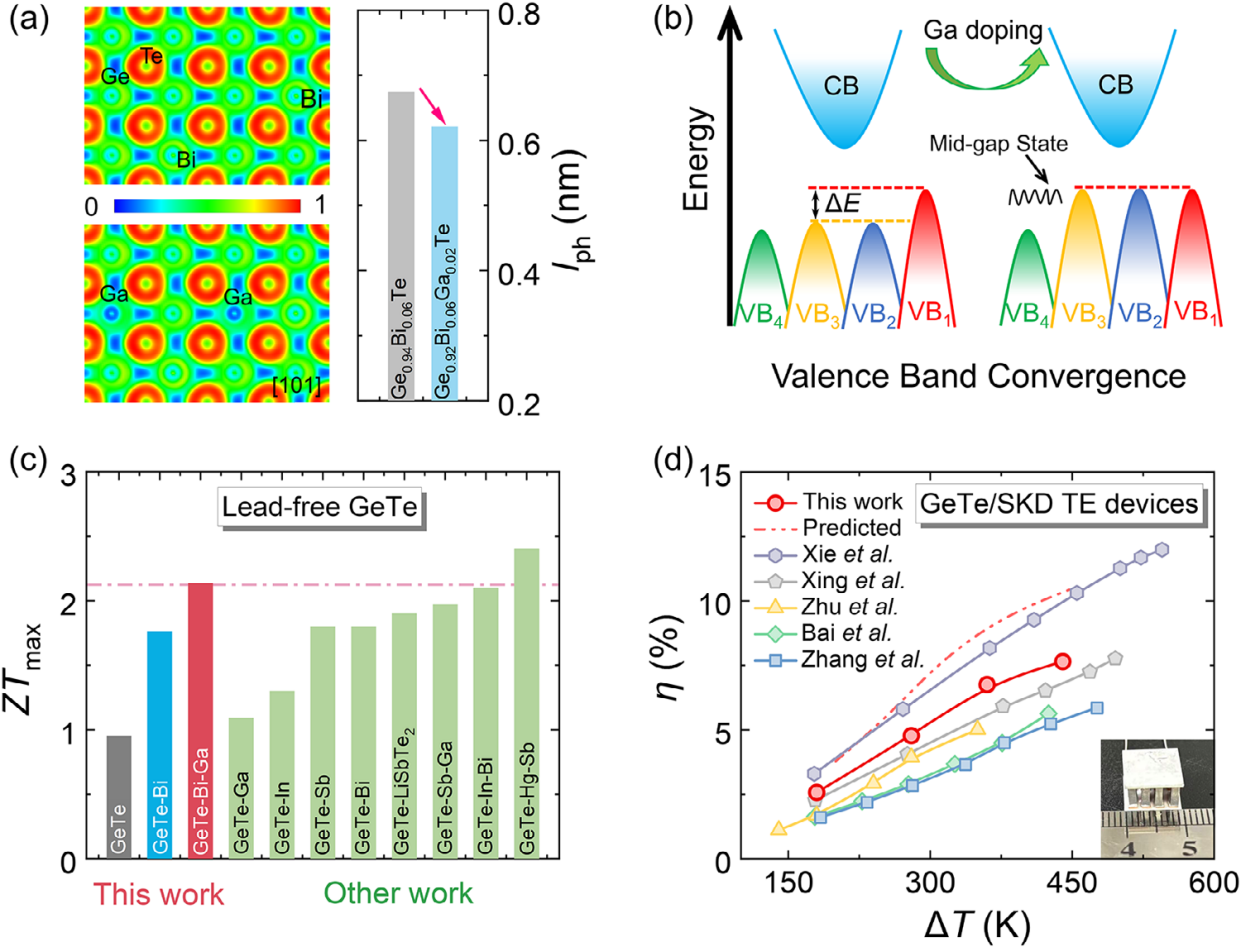
(a) Electron localization function (ELF) maps of *r*‐Ge_60_Bi_4_Te_64_ and *r*‐Ge_58_Bi_4_Ga_2_Te_64_, together with the calculated phonon mean free path (*l*_ph_) for Ge_0.94_Bi_0.06_Te and Ge_0.92_Bi_0.06_Ga_0.02_Te. (b) Schematic illustration of the Ga‐induced evolution of the electronic band structure in Ge_0.94_Bi_0.06_Te, where CB and VB denote the conduction band and valence band, respectively, and Δ*E* represents the energy separation among VB_1_, VB_2,_ and VB_3_. (c) Comparison of the maximum *ZT* values achieved in this work with those of previously reported lead‐free GeTe‐based TE materials [18, 19, 31, 40, 46, 47, 48, 49]. (d) Comparison of *η* as a function of Δ*T* for reported GeTe/skutterudite (SKD)‐based TE devices [35, 40, 50, 51, 52].

In this study, we incorporate Ga into the Ge_0.94_Bi_0.06_Te system to simultaneously regulate electronic and thermal transport properties. This strategy builds upon the successful optimization of *n*_H_ via Bi counter‐doping. Our analysis of the electron localization function (ELF) shows that Ga‐Te bonds exhibit less electron localization than Ge‐Te bonds, indicating a lower bond dissociation energy and weakened covalent bonding strength. This bond softening enhances lattice vibrational amplitudes and intensifies phonon scattering, thereby shortening the phonon mean free path (*l*_ph_, calculated according to Equation S1 and table S1) and effectively suppressing *κ*_lat_ (Figure 1a). Concurrently, the hybridization between Ga *s* orbitals and Te *p* orbitals induces a mid‐gap electronic state and significantly reduces Δ*E* between the light and heavy VB extrema, leading to pronounced band convergence. This electronic structure modulation simultaneously enhances the density‐of‐state effective mass (*m* ^*^) and preserves favorable carrier transport characteristics, as evidenced by the improved electrical performance (Figure 1b). As a result of the synergistic optimization of band structure and phonon transport, a high *ZT* exceeding 2.1 is achieved at 653 K, representing competitive performance among lead‐free GeTe‐based TE materials (Figure 1c) [18, 19, 31, 40, 46, 47, 48, 49]. Furthermore, a corresponding single‐stage TE device delivers an experimental *η* of 7.7% under a temperature difference (Δ*T*) of 440 K, surpassing most reported GeTe/skutterudite (SKD)‐based TE devices (Figure 1d) [35, 40, 50, 51, 52].

## 2 Result and Discussion

A series of *p*‐type polycrystalline Ge_0.94‐x_Bi_0.06_Ga_x_Te (x = 0‐0.03) ingots were synthesized using a conventional solid‐state melting method. Room‐temperature powder x‐ray diffraction (PXRD) patterns of all samples can be well indexed to rhombohedral (*r*‐) GeTe (PDF#47‐1079). In addition, a small number of weak diffraction peaks corresponding to elemental Ge (PDF#04‐0545) are observed (Figure S1), which can be attributed to thermodynamically driven Ge cation vacancies and is commonly reported in GeTe‐based materials [18, 38].

**FIGURE 2:**
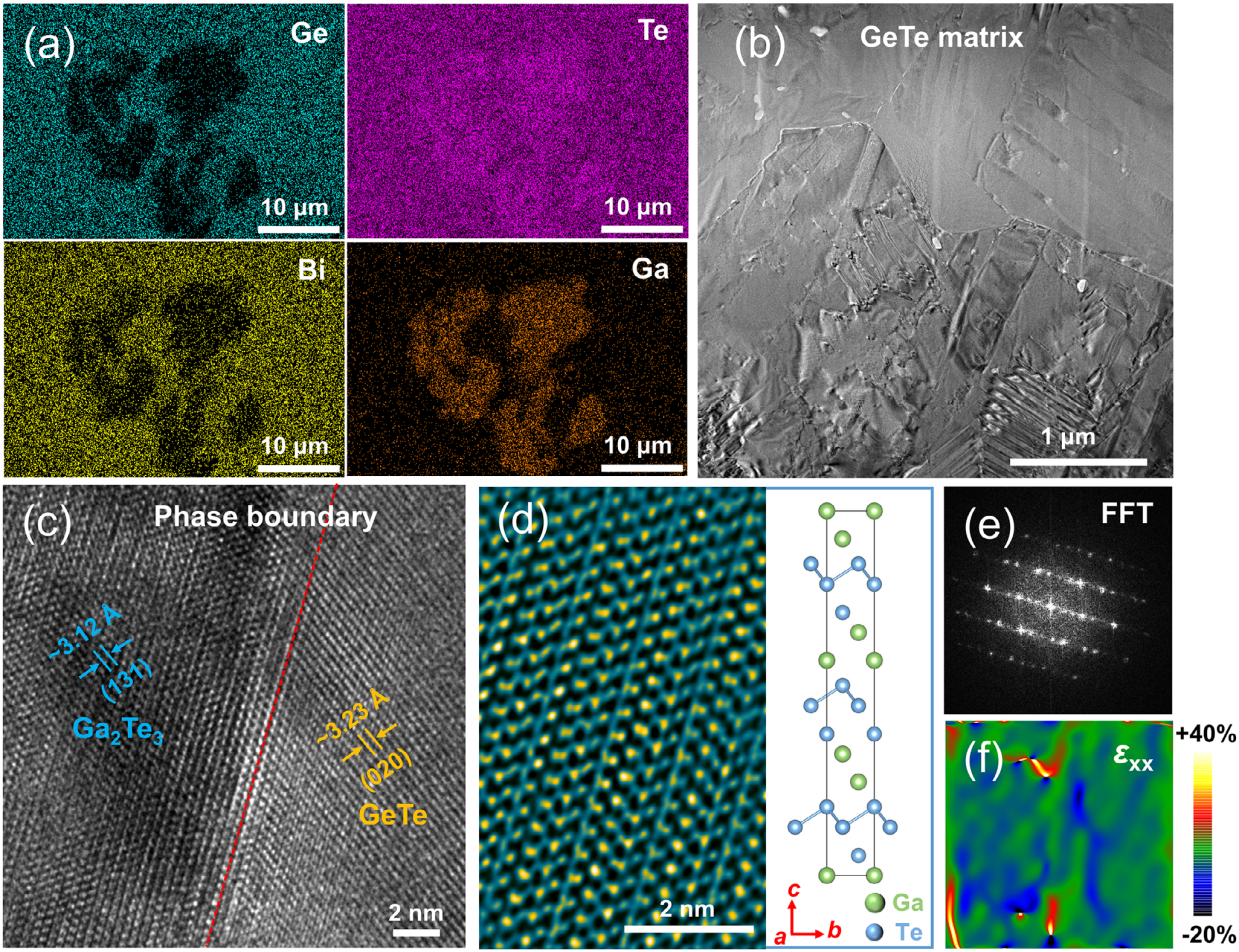
Microstructural characterization of Ge_0.92_Bi_0.06_Ga_0.02_Te. (a) Elemental mapping reveals the presence of Ga_2_Te_3_ nanoprecipitates embedded in the GeTe matrix. (b) Low‐magnification STEM image showing the microstructural features of the GeTe matrix. (c) HRTEM image of the Ga_2_Te_3_/GeTe heterointerface. (d) HRTEM image and corresponding atomic structure model of the Ga_2_Te_3_ phase. (e) Fast Fourier transform (FFT) images patterns corresponding to the region in (c). (f) Geometric phase analysis (GPA) strain mapping derived from (c), illustrating the local lattice distortion at the Ga_2_Te_3_/GeTe interface.

To elucidate the micro‐/nanostructural features and crystallographic characteristics, scanning electron microscopy (SEM) combined with back‐scattered electron (BSE) imaging was employed. For the undoped sample (x = 0), randomly distributed micro‐/nanoscale Ge precipitates are observed (Figure S2), in good agreement with the XRD results. Upon Ga incorporation, distinct microstructural evolution occurs. In the x = 0.02 sample, BSE imaging and the corresponding energy‐dispersive spectroscopy (EDS) analysis reveal the formation of Ga‐Te compound precipitates, which can be readily distinguished from the GeTe matrix by their pronounced dark contrast (Figure 2a; Figure S3).

To gain atomic‐scale insight, scanning transmission electron microscopy (STEM) investigations were further performed on the x = 0.02 sample. Low‐magnification STEM images reveal a high density of lattice distortions, grain boundaries, and periodic herringbone‐like ferroelectric domain structures with alternating contrast throughout the matrix (Figure 2b; Figure S4). Evidence of strong strain and compositional inhomogeneity is also found in the form of localized Ge vacancy clusters and nanoscale mass fluctuations(Figure S5). Figure 2c presents a high‐resolution STEM image of the interface between a Ga‐Te precipitate and the GeTe matrix. Combined atomic‐resolution imaging and EDS elemental mapping confirm that the precipitates correspond to the Ga_2_Te_3_ phase, exhibiting an interplanar spacing of ∼3.12 Å associated with the (131) crystallographic plane (Figure 2d). Dislocation networks and lattice distortions are visible at the Ga_2_Te_3_/GeTe heterointerface (Figures 2e; Figure S6), which geometric phase analysis confirms are the source of significant local strain fluctuations(Figure 2f). These hierarchical defect structures are expected to play a critical role in modulating phonon transport, as discussed in the following sections.

**FIGURE 3:**
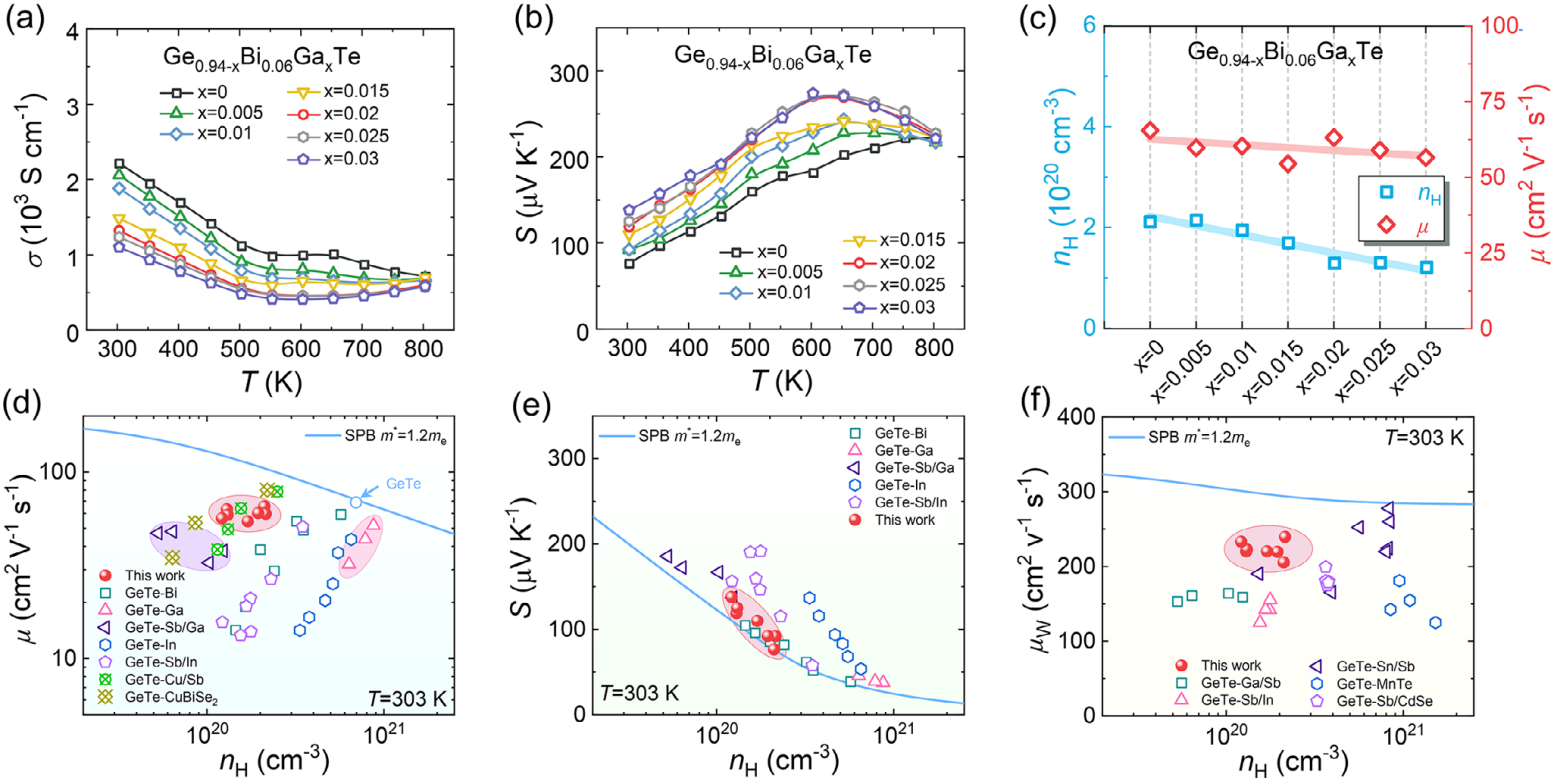
Electrical transport properties of Ge_0.94‐x_Bi_0.06_Ga_x_Te. Temperature‐dependent (a) *σ* and (b) *S*. (c) Room‐temperature *n*_H_ and *µ*. (d‐f) Experimentally measured *S*, *µ*, and weighted mobility *µ_W_* as functions of *n*_H_ at 303 K, compared with the theoretically calculated Pisarenko curves based on the single parabolic band (SPB) model.

Figure 3 illustrates the electrical transport properties of Ge_0.94‐x_Bi_0.06_Ga_x_Te (x = 0–0.03). As shown in Figure 3a, all samples exhibit a negative temperature dependence of *σ*, characteristic of degenerate semiconductors. With increasing Ga content, *σ* gradually decreases, from 2217 S cm^−1^ for the x = 0 sample to 1321 S cm^−1^ for the x = 0.03 sample at 303 K. In contrast, Ga doping markedly enhances *S* over the entire temperature range investigated (Figure 3b). With Ga doping, the Seebeck coefficient rises above 270 µV·K^−1^ at 603 K. This enhancement signifies a successful optimization of the interplay between *n*_H_ and band structure, leading to superior electronic transport properties for high‐efficiency thermoelectrics.

The concurrent evolution of *σ* and *S* with Ga content is well correlated with the variation in *n*_H_ and mobility (*µ*) measured at room temperature (Figure 3c). The incorporation of Ga suppresses Ge vacancy defects, thereby significantly reducing *n*_H_ in Ge_0.94‐x_Bi_0.06_Ga_x_Te alloys. Remarkably, despite the introduction of alloying elements and associated point defects, *µ* does not exhibit a substantial degradation upon Ga incorporation. This behavior can be rationalized by two competing effects. The reduced electronic localization associated with Ga‐Te bonding (Figure 1a), together with the small lattice mismatch at the Ga_2_Te_3_/GeTe heterointerfaces (Figure 2d), effectively mitigates carrier scattering and helps preserve high *µ*. Meanwhile, Ga substitution at Ge sites and the accompanying point defect scattering tend to reduce *µ*. The interplay between these opposing factors results in a slight net decrease in *µ*. Importantly, the resulting *µ* remains higher than those reported for most Bi‐, Ga‐, and In‐doped GeTe‐based alloys [19, 40, 46, 47, 53] and is even comparable to that of high *µ* systems such as GeTe‐Cu/Sb and GeTe‐CuBiSe_2_ [54, 55], as listed in (Figure 3d). This favorable transport behavior highlights the effectiveness of Ga‐induced band and bonding modulation in maintaining high *µ* while optimizing *n*_H_.

To understand the origin of *S* enhancement induced by Ga doping in Ge_0.94‐x_Bi_0.06_Ga_x_Te, the experimentally measured room‐temperature *S*‐*n*_H_ relationship was analyzed in comparison with the theoretically calculated Pisarenko plot (Figure 3e). Given the presence of multiple valence bands in GeTe‐based alloys, a single parabolic band (SPB) model was employed to provide a simplified yet effective framework for quantitative analysis. The experimental *S*‐*n*_H_ results for the undoped sample (x = 0) fit the Pisarenko curve for *m* ^*^ = 1.2 *m*_e_ quite well. In the Ga‐doped Ge_0.94‐x_Bi_0.06_Ga_x_Te samples, however, the data deviate noticeably upward from this calculated line. This suggests that adding Ga increases the effective mass. This result corroborates the SPB model extraction at 303 K (Figure S7) and confirms that the electronic band structure undergoes significant changes due to Ga doping.

The combination of increased *m* ^*^ and high *µ* generates a marked rise in weighted mobility (*µ_W_*), calculated as *µ_W_* = *µ*(*m* ^*^/*m*_e_)^3/2^, where *m*_e_ is the free electron mass [56]. Figure S8a shows that Ge_0.94‐x_Bi_0.06_Ga_x_Te exhibits vastly improved *µ_W_*, proving the value of simultaneously manipulating carrier scattering and electronic bands. Consequently, within the optimal *n*_H_ window for GeTe‐based thermoelectrics [19, 57], the *µ_W_* values achieved in this work significantly exceed those reported for most state‐of‐the‐art GeTe‐based materials (Figure 3f) [36, 58, 40, 53, 29]. This advantage directly translates into superior electrical transport performance. Consistent with this trend, the *PF* of Ge_0.94‐x_Bi_0.06_Ga_x_Te increases monotonically with Ga content, rising from 13.0 µW cm^−1^ K^−2^ for the x = 0 sample to 20.7 µW cm^−1^ K^−2^ for the x = 0.03 sample, corresponding to an enhancement of approximately 59% (Figure S8b).

**FIGURE 4:**
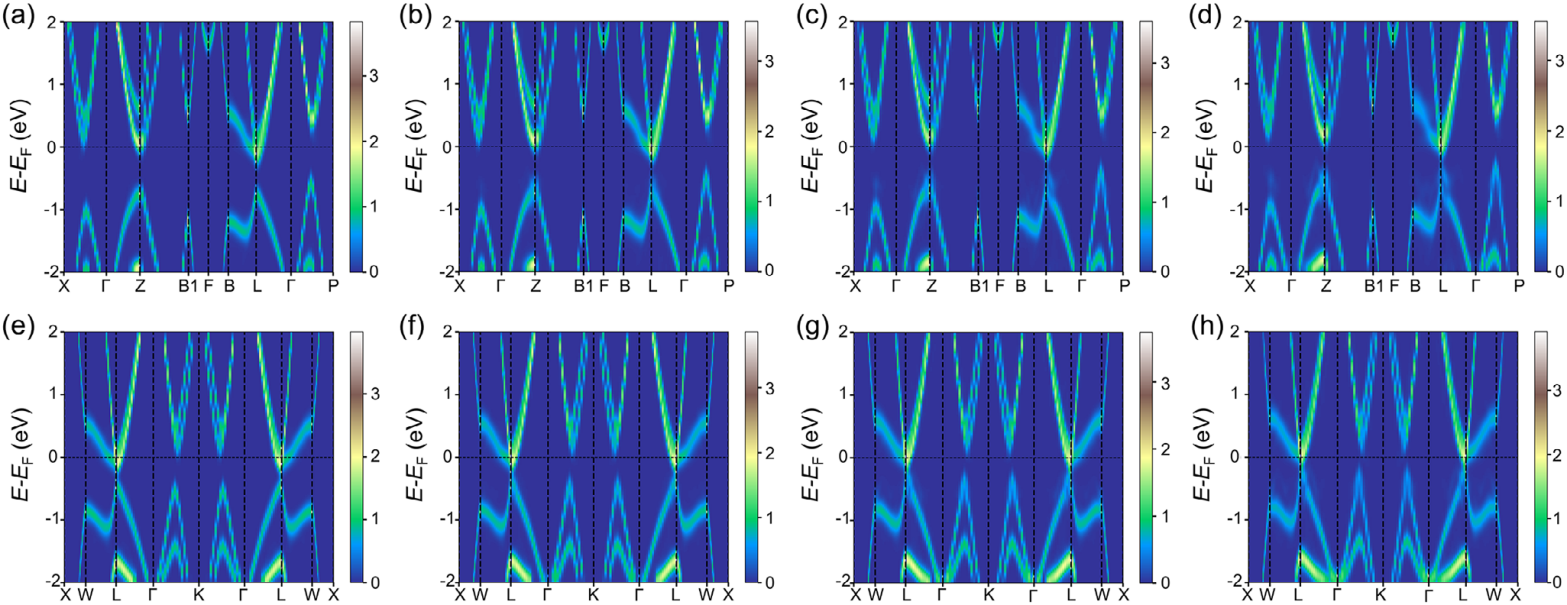
Ga‐induced evolution of the electronic band structure of Ge_0.94_Bi_0.06_Te. (a‐d) Calculated electronic band structures of rhombohedral GeTe with increasing Ga content: (a) *r‐*Ge_60_Bi_4_Te_64_, (b) *r‐*Ge_59_Bi_4_GaTe_64_, (c) *r‐*Ge_58_Bi_4_Ga_2_Te_64_, and (d) *r‐*Ge_57_Bi_4_Ga_3_Te_64_. (e‐h) Corresponding calculated electronic band structures of the cubic phases: (e) *c‐*Ge_60_Bi_4_Te_64_, (f) *c‐*Ge_59_Bi_4_GaTe_64_, (g) *c‐*Ge_58_Bi_4_Ga_2_Te_64_, and (h) *c‐*Ge_57_Bi_4_Ga_3_Te_64_.

To explore the mechanism underlying the *S* enhancement induced by Ga doping, we constructed a rhombohedral 4×4×4 supercell of Ga‐doped Ge_0.94_Bi_0.06_Te, incorporating spin‐orbital coupling (SOC) and band unfolding techniques (Figure S9). The scale bars on the right indicate the spectral weight function (Figure 4). The pristine *r‐*Ge_60_Bi_4_Te_64_ supercell is an indirect‐bandgap semiconductor with multiple valence band extrema and relatively small Δ*E*, whereas the corresponding cubic phase (*c‐*Ge_60_Bi_4_Te_64_) exhibits a direct bandgap, consistent with previous reports [28]. Ga incorporation elevates the VB edge energies near the *Z* and *L* points, reducing the Δ*E* between these levels and the VB edges along the *Γ‐P* direction. With increasing Ga content, Δ*E* decreases further, thereby enhancing band convergence. Furthermore, Ga doping induces splitting of the VB edges along the *Γ‐X* direction, generating new energy bands that expand toward the Fermi level as the doping concentration increases. Density of states analysis reveals clear hybridization between Ga *s* orbitals and the *p* orbitals of Ge and Te, giving rise to a mid‐gap state (Figure S10). Collectively, the multi‐valent band convergence and mid‐gap state induced by Ga doping contribute to the enhanced *m* ^*^ and improved electronic transport properties.

**FIGURE 5:**
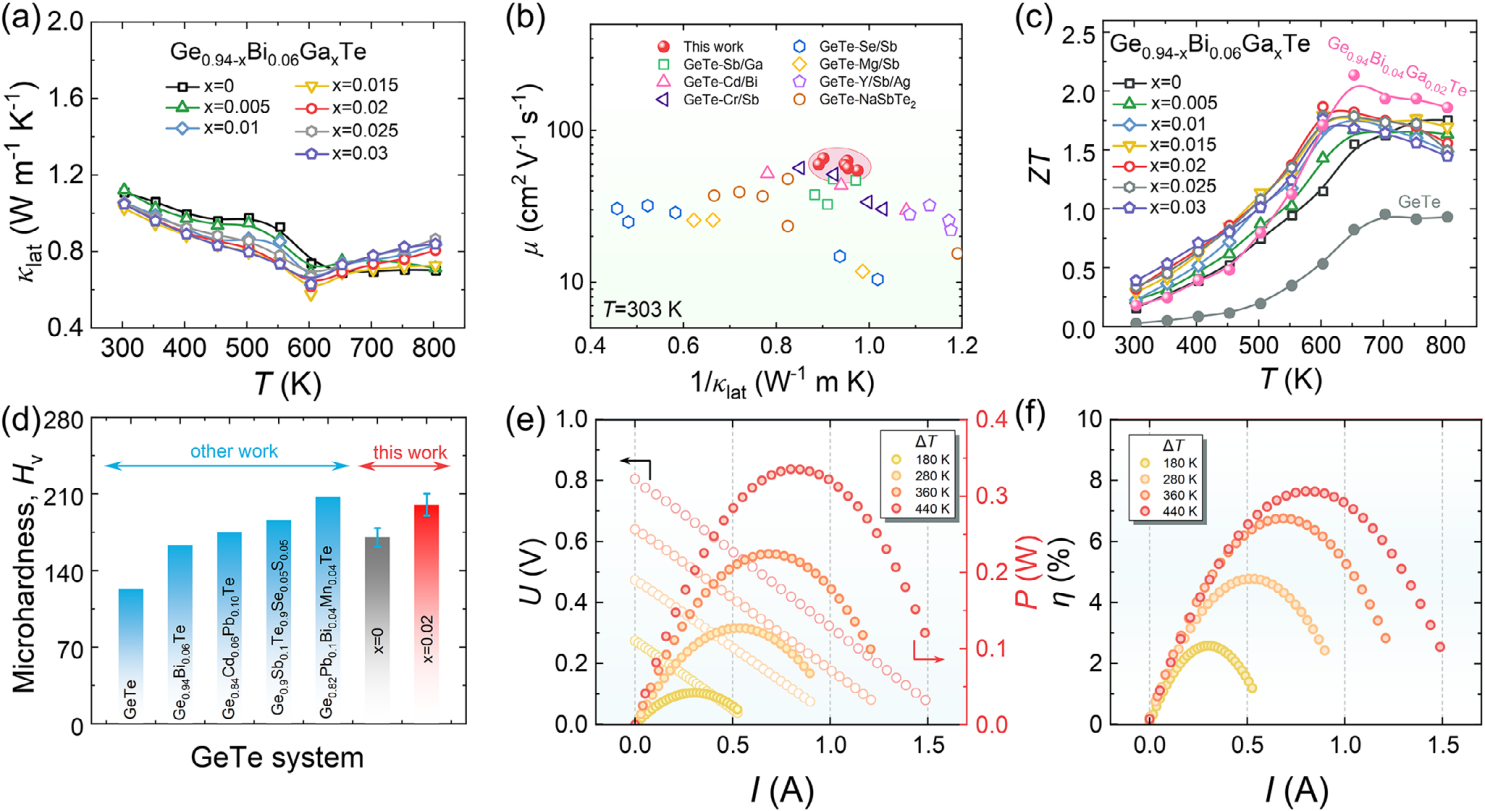
Thermal transport, TE performance, mechanical robustness, and device‐level characteristics. (a) Temperature‐dependent *κ*_lat_ of Ge_0.94‐x_Bi_0.06_Ga_x_Te. (b) *µ* as a function of 1/*κ*_lat_ at 303 K, highlighting the trade‐off between electrical and phonon transport. (c) Temperature‐dependent *ZT* of Ge_0.94‐x_Bi_0.06_Ga_x_Te. (d) Comparison of room‐temperature Vickers microhardness of Ge_0.94‐x_Bi_0.06_Ga_x_Te with reported GeTe‐based TE materials [26, 63, 64, 65, 67]. (e,f) Measured *U* and *P* (e) and *η* (f) as functions of *I* under various Δ*T* values for the fabricated 7‐pair TE device.

Figure S11 presents the temperature‐dependent *κ* of Ge_0.94‐x_Bi_0.06_Ga_x_Te, which decreases with increasing Ga content, reaching a minimum value of 1.63 W m^−1^ K^−1^ at 303 K for the x = 0.03 sample. The *κ*_ele_ was calculated using the Wiedemann‐Franz law, and the *κ*_lat_ was obtained by subtracting *κ*_ele_ from *κ* (Figure 5a). The *κ*_lat_ of the Ge_0.94‐x_Bi_0.06_Ga_x_Te exhibits a minimum of 1.02 W m^−1^ K^−1^ at 303 K, further decreasing to 0.57 W m^−1^ K^−1^ at 603 K, approaching the theoretical minimum (∼0.5 W m^−1^ K^−1^) predicted by Cahill's model [59]. This pronounced reduction in *κ*_lat_ can be attributed to the synergistic enhancement of phonon scattering via two mechanisms. First, the formation of Ga‐Te bonds weakens the Ge‐Te covalent interactions, increasing the lattice vibration disorder (Figure 1a). Second, the introduced hierarchical nanostructural defects, including lattice distortion, domain structure, Ge vacancy clusters, and nano‐heterointerfaces, effectively scatter phonons over a wide frequency range (Figure 2). Together, these effects substantially suppress *κ*_lat_, contributing to the high TE performance of the Ga‐doped GeTe system.

Chemical doping effectively reduces the inherently high *n*_H_ in pristine GeTe; however, it is often accompanied by a decline in *µ*. Figure 5b compares *µ* as a function of 1/*κ*_lat_ for various *Q*‐doped GeTe TE material at 303 K (*Q* = Sb/Ga [40], Cd/Bi [38], Cr/Sb [28], Se/Sb [24], Mg/Sb [60], Y/Sb/Ag [61], NaSbTe_2_ [62]). Notably, the *µ*/*κ*_lat_ values of Ge_0.94‐x_Bi_0.06_Ga_x_Te samples are superior to those of previously reported systems, which is crucial for achieving synergistic optimization of TE transport properties. Consequently, the x = 0.02 sample exhibits a peak *ZT* of 1.9 and an average *ZT* (*ZT*_ave_) of 1.3 (Figure 5c). Further tuning of the Bi content allows the peak *ZT* to exceed 2.1, representing a significant improvement over the pristine GeTe. In addition to the transport performance, Ga‐induced evolution of crystal defects enhances the mechanical properties. The room‐temperature Vickers microhardness (*H*_v_) increases from 170.4 *H*_v_ for the x = 0 sample to 200.2 *H*_v_ for the x = 0.02 sample, corresponding to a 17% improvement, which is competitive among reported GeTe‐based thermoelectrics (Figure 5d) [26, 63, 64, 65]. This mechanical performance enhancement is attributed to the in‐situ precipitation of Ga_2_Te_3_ nanoprecipitates and the nanointerface formed with the matrix, which effectively pins dislocation slip and suppresses crack propagation [66].

To assess the practical power generation potential of the optimized *p*‐type Ge_0.94_Bi_0.04_Ga_0.02_Te, a unipolar 7‐pair TE device was fabricated in combination with an *n*‐type filled skutterudite material (Yb_0.35_Ga_0.2_Co_4_Sb_12.05_) (Figure S12). The device dimensions are 10 mm × 10 mm × 4.5 mm, with *n*‐ and *p*‐type leg cross sections of 1.4 mm × 1.4 mm. The cold‐side temperature (*T*_c_) was maintained at 303 K, while the hot‐side temperature (*T*_h_) was varied to evaluate power generation performance. The measured output voltage (*U*) and current (*I*) exhibit excellent linearity under various temperature differences (Δ*T* = *T*_h_‐*T*_c_), where the slope and intercept correspond to the internal resistance (*R*_in_) and open‐circuit voltage (*U*_oc_) of the device, respectively (Figure 5e). For a given Δ*T*, the output power (*P*) initially increases with *I*, reaches a maximum when the external load matches *R*_in_, and then decreases. Figure 5f presents the measured *η* as a function of Δ*T*. A maximum *η* of 7.7% is achieved at Δ*T* = 440 K, demonstrating the considerable potential of this TE device for efficient waste heat harvesting.

## 3 Conclusion

In summary, we have achieved simultaneous optimization of the TE performance of Ge_0.94_Bi_0.06_Te through Ga doping, attaining peak *ZT* values exceeding 2.1. Ga incorporation effectively promotes convergence of the three VB edges, while the hybridization between Ga *s* orbitals and Te *p* orbitals generates mid‐gap states, collectively enhancing *m* ^*^ and electrical transport properties. In parallel, the formation of Ga‐Te bonds weakens the Ge‐Te covalent interactions and amplifies the lattice vibrations, and the introduction of hierarchical lattice defects further suppresses *κ*_lat_. For practical demonstration, a single‐stage, 7‐pair, lead‐free TE device was fabricated using Ge_0.94_Bi_0.04_Ga_0.02_Te as the *p*‐type leg and Yb_0.35_Ga_0.2_Co_4_Sb_12.05_ as the *n*‐type leg, achieving a *η* of 7.7% at Δ*T* = 440 K. This work demonstrates that the synergistic tuning of the electronic structure and chemical bonding provides an effective route to weaken electron‐phonon coupling, offering valuable insights into the design, performance enhancement, and mechanistic understanding of other high‐performance TE materials and devices.

## Conflicts of Interest

The authors declare no conflicts of interest.

## Supporting information




**Supporting File**: advs74427‐sup‐0001‐SuppMat.docx.

## Data Availability

The data that support the findings of this study are available from the corresponding author upon reasonable request.
