# Supplementary material for: Balancing Effective Mass and Carrier Mobility via Ga‐Induced Multi‐Band Valley Engineering in GeTe Thermoelectrics
Source: Adv Sci (Weinh). 2026 Feb 15;13(24):e74427. doi: 10.1002/advs.74427 (PMC13116235; doi:10.1002/advs.74427)
Supplement: Supplementary file 1 — Supporting File: advs74427‐sup‐0001‐SuppMat.docx. [file ADVS-13-e74427-s001.docx]

**Supplementary materials**

**Balancing Effective Mass and Carrier Mobility via Ga-Induced Multi-Band Valley Engineering in GeTe Thermoelectrics**

Jianglong Zhu, Yan Zhong, Xiang An, Huangshui Ma, Jie Zheng, Huagang Xiao, Shiyuan Zhan, Pingan Song, Min Hong,^*^ and Ran Ang^*^

J. L. Zhu, H. G. Xiao

College of Physics, Chengdu University of Technology, Chengdu 610059, China.

Y. Zhong

School of Intelligent Manufacturing, Sichuan University of Arts and Science, Dazhou 635000, China.

S. Y. Zhan

College of Energy, Chengdu University of Technology, Chengdu 610059, China.

H. Ma, P. Song, M. Hong

Centre for Future Materials, School of Science, Engineering and Digital Technologies, University of Southern Queensland, Springfield Campus, QLD 4300, Australia

E-mail: [min.hong@unisq.edu.au](mailto:min.hong@unisq.edu.au)

X. An, J. Zheng, R. Ang

Key Laboratory of Radiation Physics and Technology, Ministry of Education, Institute of Nuclear Science and Technology, Sichuan University, Chengdu 610064, China

E-mail: [rang@scu.edu.cn](mailto:rang@scu.edu.cn)

**Supplementary Methods**

**1. Synthesis of Ge_0.94-x_Bi_0.06_Ga_x_Te (x=0-0.03)**

High-purity elemental Ge, Te, Bi, and Ga (99.999%) were weighed according to the nominal compositions of Ge_0.94-x_Bi_0.06_Ga_x_Te (x=0-0.03). The starting materials were loaded into quartz ampoules, evacuated to ~10⁻⁴ Torr, and then sealed. The sealed ampoules were heated to 1123 K in a muffle furnace and maintained at this temperature for 12 h to ensure complete melting and homogenization, followed by an additional dwell at 1123 K for 6 h. Subsequently, the ampoules were rapidly quenched in ice water to retain the high-temperature phase. The quenched ingots were annealed at 973 K for 72 h to promote compositional uniformity and structural equilibration. The annealed ingots were then pulverised into fine powders and consolidated by vacuum hot pressing at 873 K under an axial pressure of ~80 MPa for 40 min, yielding dense polycrystalline bulk samples.

**2. Structural characterisation**

Phase identification was carried out by powder X-ray diffraction (XRD) using Cu K*α* radiation (*λ* = 1.5418 Å). The microstructural features were examined by field-emission scanning electron microscopy (FE-SEM, JEOL JSM-7100) and transmission electron microscopy (TEM, FEI Tecnai F20). High-resolution TEM (HRTEM) and scanning transmission electron microscopy (STEM) observations were performed to analyse the crystal structure and interfacial characteristics at the nanoscale. Elemental composition and spatial distribution were analysed using an energy-dispersive X-ray spectroscopy (EDS) system integrated with the TEM.

**3. Transport property measurements**

The electrical transport properties, including electrical conductivity (*σ*) and Seebeck coefficient (*S*), were measured simultaneously using a commercial CTApro system (Beijing Cryoall Science and Technology Co., Ltd., China). The total thermal conductivity (*κ*) was calculated according to *κ* = *ρC*_p_*D*, where *ρ* is the sample density determined from the mass-to-volume ratio, *C*_p_ is the specific heat capacity estimated using the Debye model [1], and *D* is the thermal diffusivity measured by laser flash analysis (LFA 467, Netzsch, Shanghai, China). The electronic contribution to thermal conductivity (*κ*_ele_) was estimated using the Wiedemann–Franz relation, *κ*_ele_=*Lσ*T, where the Lorenz number (*L*) was determined based on the single parabolic band (SPB) model.[2] Hall effect measurements were performed at room temperature using the Van der Pauw geometry under a magnetic field of 1.5 T. The Hall carrier concentration (*n*_H_) and mobility (*μ*) were calculated using *n*_H_=1/(*eR*) and *μ*=*σR*, respectively, where *e* is the elementary charge. The longitudinal and transverse sound velocities (*v*) were measured using an ultrasonic pulse-echo technique with a pulse receiver (Olympus-NDT) coupled to a digital oscilloscope (Keysight). Vickers microhardness (*H*_v_) was evaluated at room temperature using an HVS-1000 tester. The reported value represents the average of five independent measurements taken at different surface spots with a load of 2 N and a dwell time of 10 s.

**4. Density functional theory (DFT) calculation****s**

DFT calculations were performed using the Vienna *ab initio* simulation package (VASP),[3–5] with post-processing carried out using the VASPKIT package.[6] The electron exchange-correlation interactions were described by the Perdew-Burke-Ernzerhof (PBE) functional within the generalised gradient approximation (GGA).[7] The plane-wave basis set was employed with a kinetic energy cutoff of 450 eV. A 4×4×4 supercell of Ge_64_Te_64_ constructed from the primitive unit cell was adopted to calculate the formation energies and electronic band structures. To facilitate direct comparison with the primitive cell band dispersion, the supercell band structures were unfolded onto the high-symmetry k-paths of the primitive Brillouin zone using the BandUP code.[8,9]

**5. Thermoelectric (TE) device fabrication and characterisation**

A seven-pair TE device was fabricated using *n*-type Yb_0.35_Ga_0.2_Co_4_Sb_12.05_ and *p*-type Ge_0.94_Bi_0.04_Ga_0.02_Te as the *n*- and *p*-type legs, respectively. Before assembly, nickel layers were electroplated onto the top and bottom surfaces of the TE legs to serve as contact layers by Xianghe Oriental Electronics Co., Ltd., China. The Ni/SKD/Ni and Ni/GeTe/Ni were precision-cut to dimensions of 1.4 mm×1.4 mm×4.5 mm. These elements were arranged in an alternating p-n configuration on 10 mm×10 mm×0.65 mm alumina (Al_2_O_3_) ceramic substrates. Interconnections were established using copper bridges soldered with commercial nano-silver paste, while current supply and voltage monitoring were facilitated by copper leads attached to the cold-side plate.

Device performance, specifically output power (*P*) and conversion efficiency (*η*), was evaluated under vacuum using a self-developed measurement system [10,11] calibrated against a Mini PEM (Advance Riko, Japan). The cold-side temperature (*T*_c_) was stabilized at 303 K via a circulating cooling loop. To minimize contact thermal resistance, a composite interface of 0.2 mm graphite paper and QM850 thermal grease was applied to both the heater-device and device-cooler interfaces. Temperature gradients were monitored using K-type thermocouples (Omega), while heat flux (*Q*) passing through the device was quantified using a 10 mm×10 mm oxygen-free copper reference block. Maximum output power was identified by tuning the external load resistance, and conversion efficiency was subsequently calculated as *η* =*P*/(*P*+*Q*).

**6. Calculation of the mean free path of phonons**

Assuming that phonon scattering dominates the thermal transport, the phonon mean free path (*l*_ph_) at room temperature was estimated using the relation:

$\kappa_{lat}=\frac{1}{3}C_{p}\nu l_{ph}$ (S1)

where *l*_ph_ is the phonon mean free path, *C_p_* is the specific heat, *v* is the sound velocity (Table S1), and *κ*_lat_ is the lattice thermal conductivity. The values of *C_p_* and *v* were determined from the Debye model and ultrasonic measurements, respectively.

**7. Calculation of average power factor and average *ZT***

The average power factor (*PF*_ave_) and average *ZT* (*ZT*_ave_) over a given temperature range were calculated using the following relations:

${PF}_{ave}=\frac{\int_{T_{c}}^{T_{h}} PFdT}{T_{h}-T_{c}}$ (S2)

$ZT_{ave}=\frac{\int_{T_{c}}^{T_{h}} ZTdT}{T_{h}-T_{c}}$ (S3)

**8.** **Single parabolic band (SPB) model**

The Lorentz number (*L*) and density-of-states effective mass (*m*^*^) were estimated based on the SPB model using the following relations:

$L=\left( \frac{k_{B}}{e} \right)^{2}\left\{ \frac{(r+7/2)F_{r+5/2}(\eta)}{(r+3/2)F_{r+1/2}(\eta)}-\left[ \frac{(r+5/2)F_{r+3/2}(\eta)}{\left( r+3/2 \right)F_{r}+1/2(\eta)} \right]^{2} \right\}$ (S4)

The Seebeck coefficient *S* is given by:

$S=\pm\frac{k_{B}}{e}\left[ \eta-\frac{(r+5/2)F_{r+3/2}(\eta)}{(r+3/2)F_{r+1/2}(\eta)} \right]$ (S5)

where *η* is the reduced chemical potential, *k*_B_ is the Boltzmann constant, *e* is the electron charge, and *r* is the scattering factor.

The carrier concentration (*n*_H_) is given by:

*n*_H_$=\frac{4\pi\left( 2m^{*}k_{B}T \right)^{3/2}}{h^{3}}F_{1/2}(\eta)$ (S6)

The Fermi-Dirac integral of order *i*(*F*_i_(*η*)) can be expressed as:

$F_{i}(\eta)=\int_{0}^{\infty} \frac{\chi^{i}}{1+e^{\chi-\eta}}d\chi$ (S7)

where *h* is Planck’s constant and *r* = -1/2, assuming that acoustic phonon scattering is the dominant scattering mechanism.

**Supplementary Figures**

**
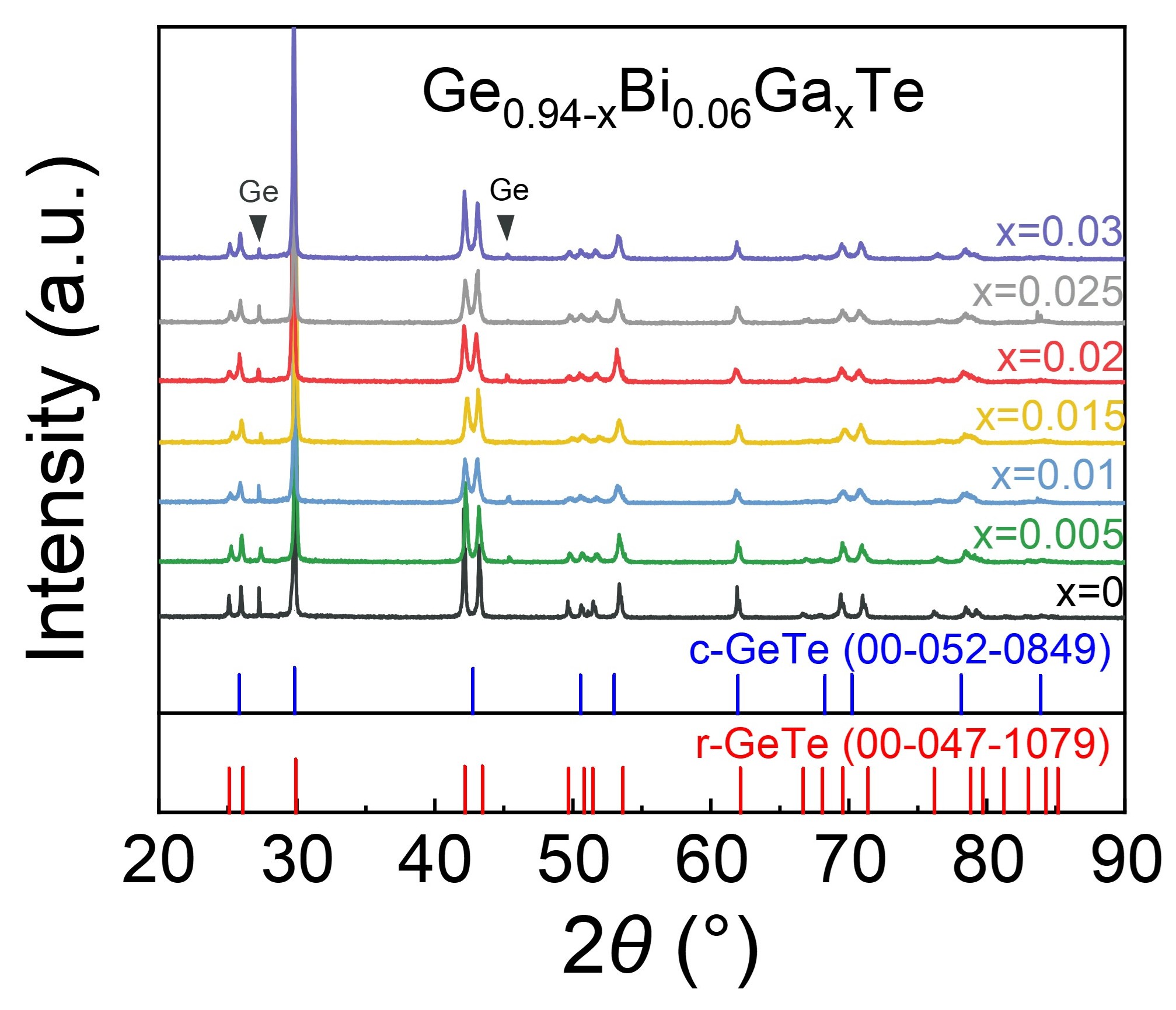
**

**Figure S1**. Room-temperature powder X-ray diffraction (XRD) patterns of Ge_0.94-x_Bi_0.06_Ga_x_Te samples, with peaks indexed to the rhombohedral GeTe phase and minor Ge secondary phases indicated.


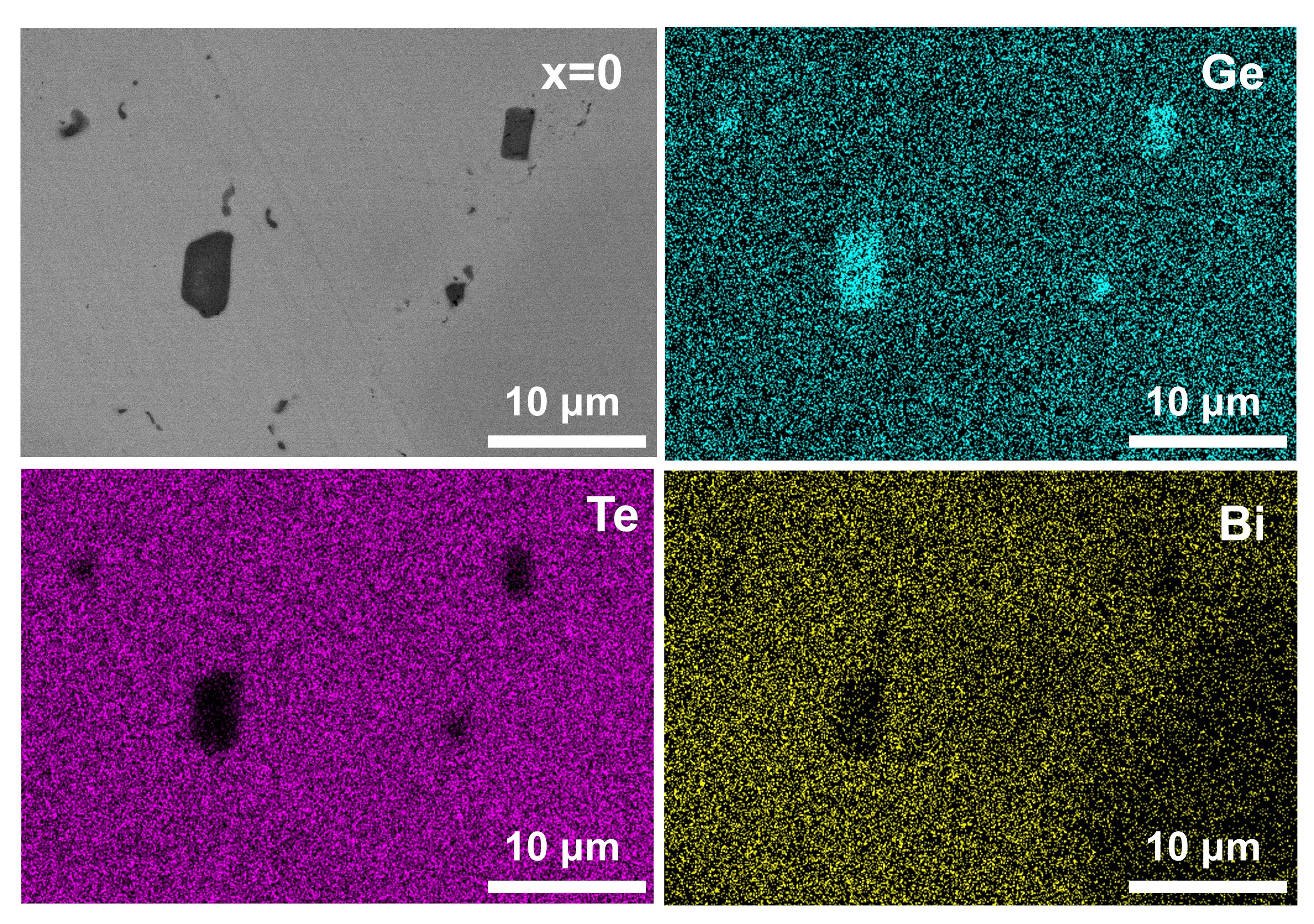


**Figure S2**. Scanning electron microscopy (SEM) image of Ge_0.94_Bi_0.06_Te and the corresponding energy-dispersive spectroscopy (EDS) mappings, showing the spatial distribution of Ge, Bi, and Te within the sample.


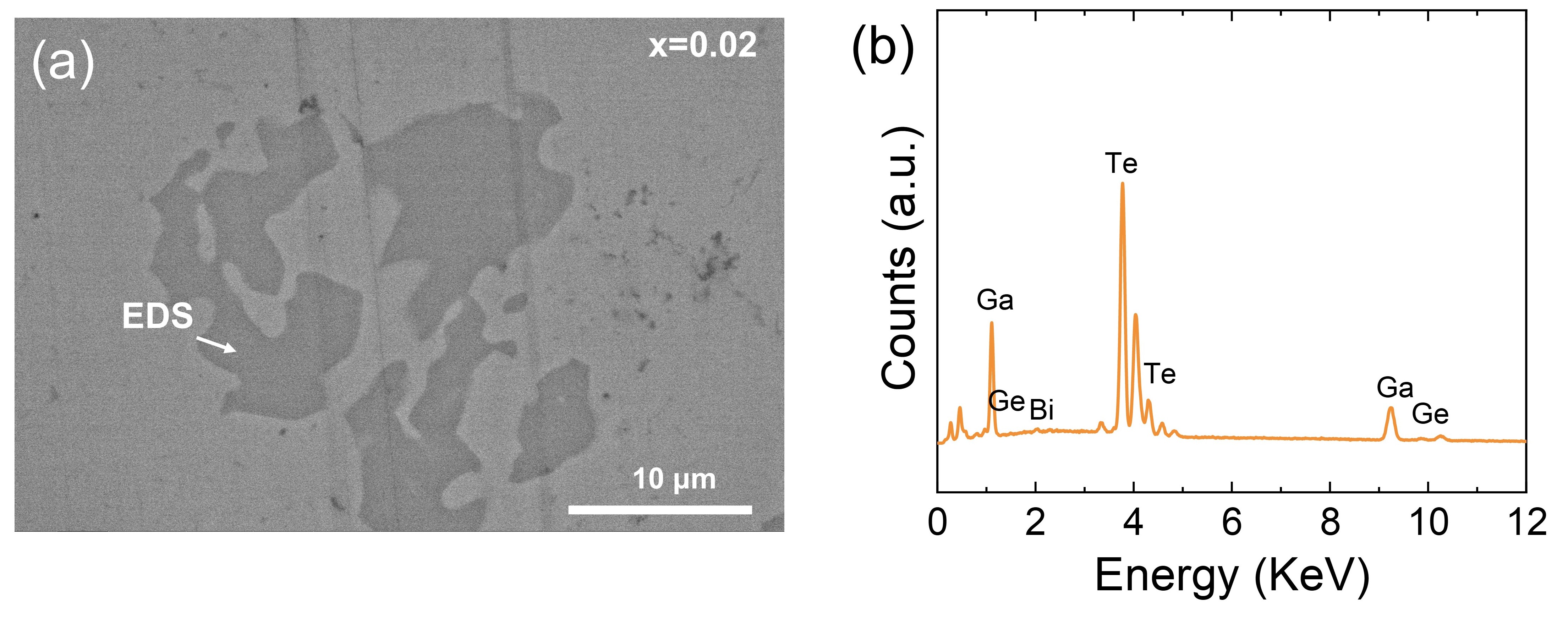


**Figure S3**. (a) SEM image of Ge_0.92_Bi_0.06_Ga_0.02_Te, highlighting the presence of precipitates. (b) The corresponding EDS spectrum of the precipitate indicated in (a), confirming its elemental composition.


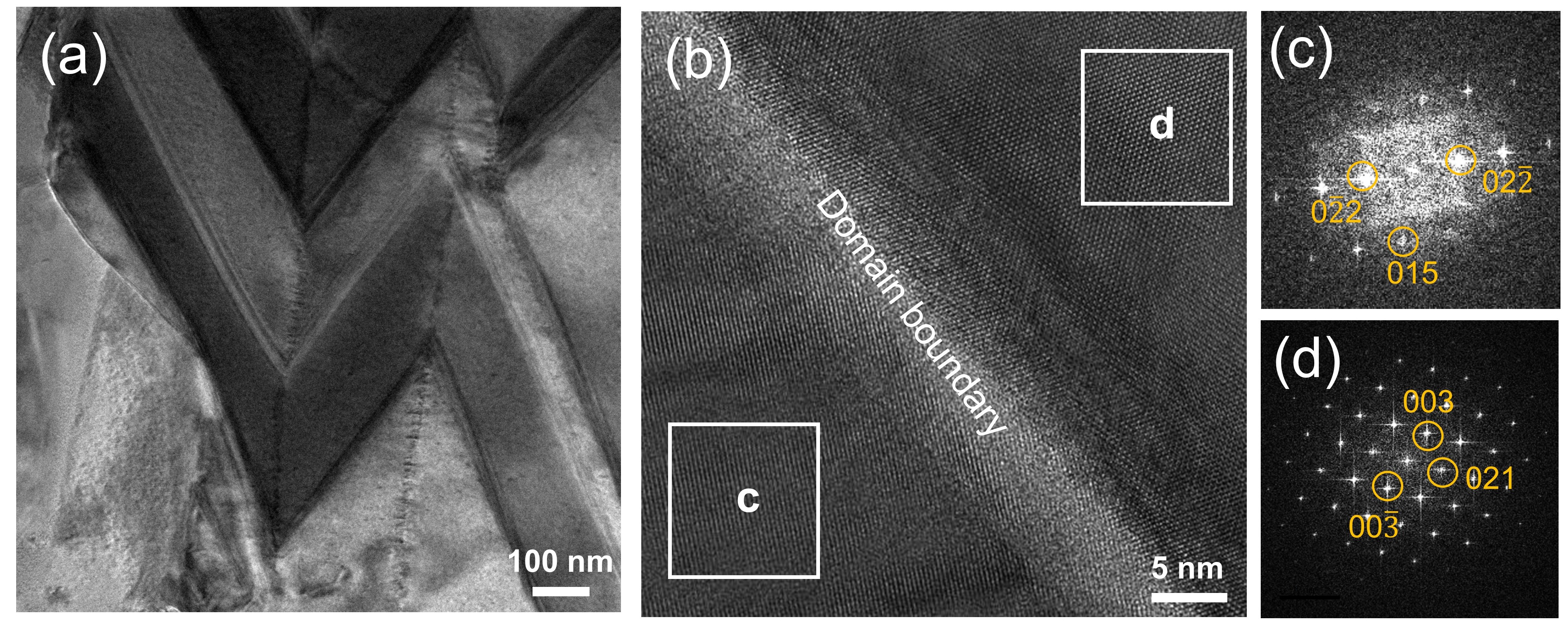


**Figure S4**. High-resolution transmission electron microscopy (HRTEM) image of Ge_0.92_Bi_0.06_Ga_0.02_Te (x=0.02), showing the periodic herringbone domain structures within the matrix.


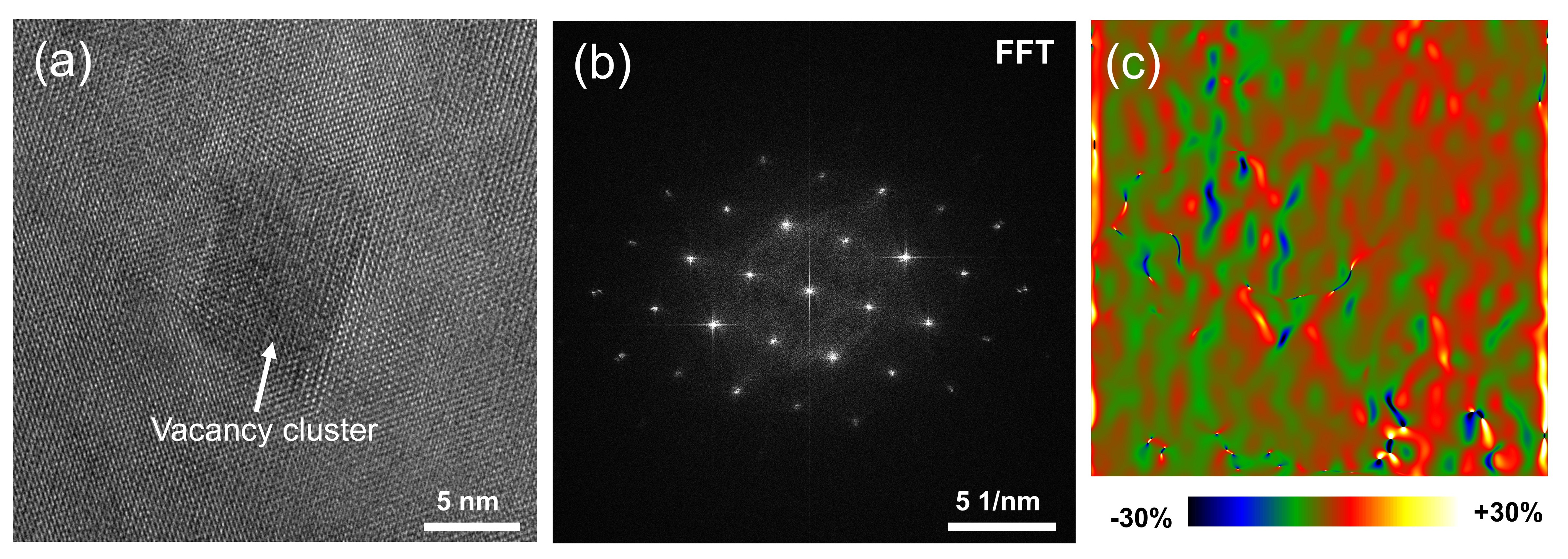


**Figure S5**. High-resolution scanning transmission electron microscopy (HR-STEM) image of Ge_0.92_Bi_0.06_Ga_0.02_Te (x=0.02), highlighting the presence of localised Ge vacancy clusters within the matrix.


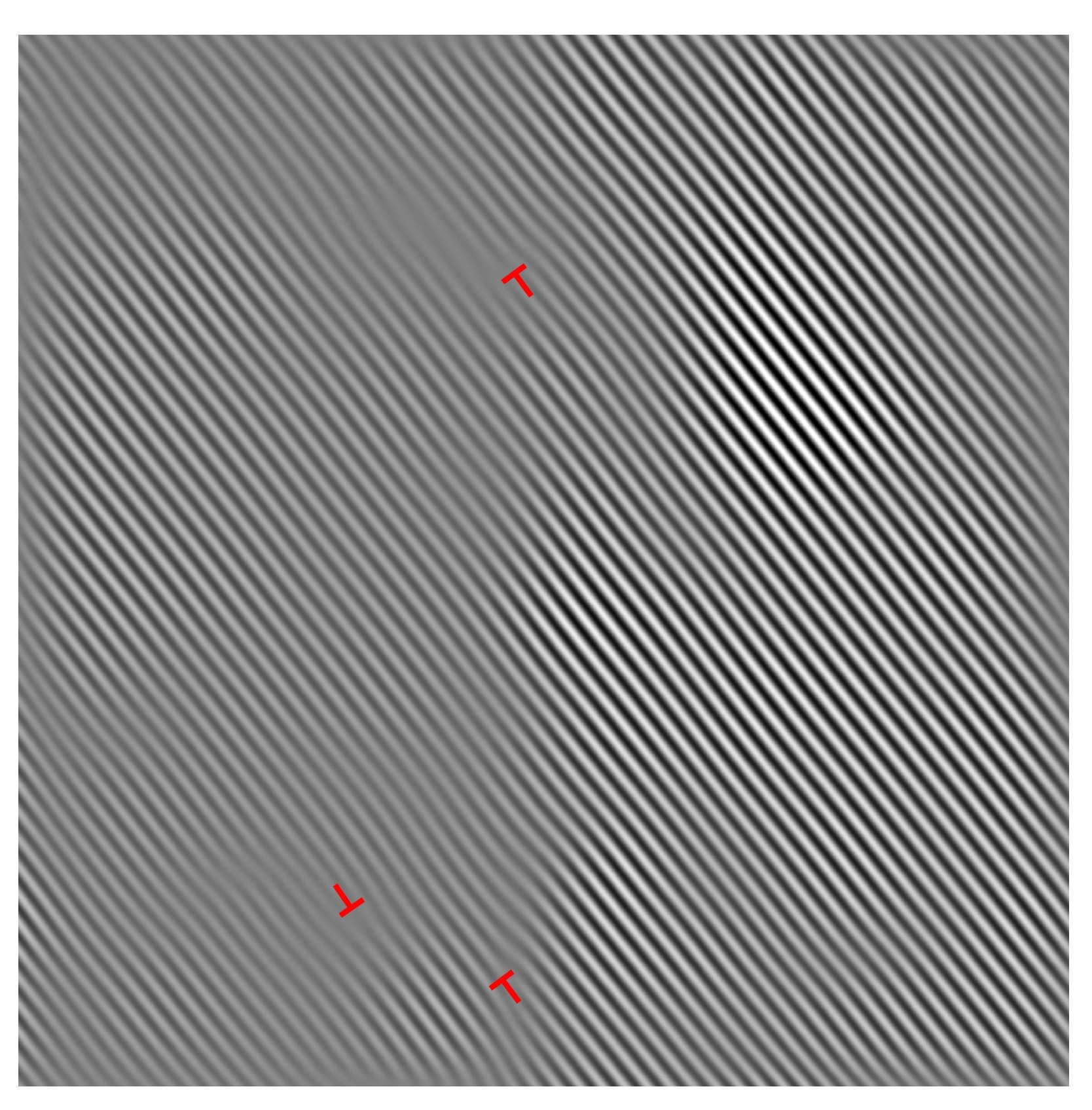


**Figure S6**. Inverse fast Fourier transform (IFFT) image of Ge_0.92_Bi_0.06_Ga_0.02_Te, emphasising the atomic-scale interfacial structure at the Ga_2_Te_3_-GeTe phase boundary shown in Figure 2e.


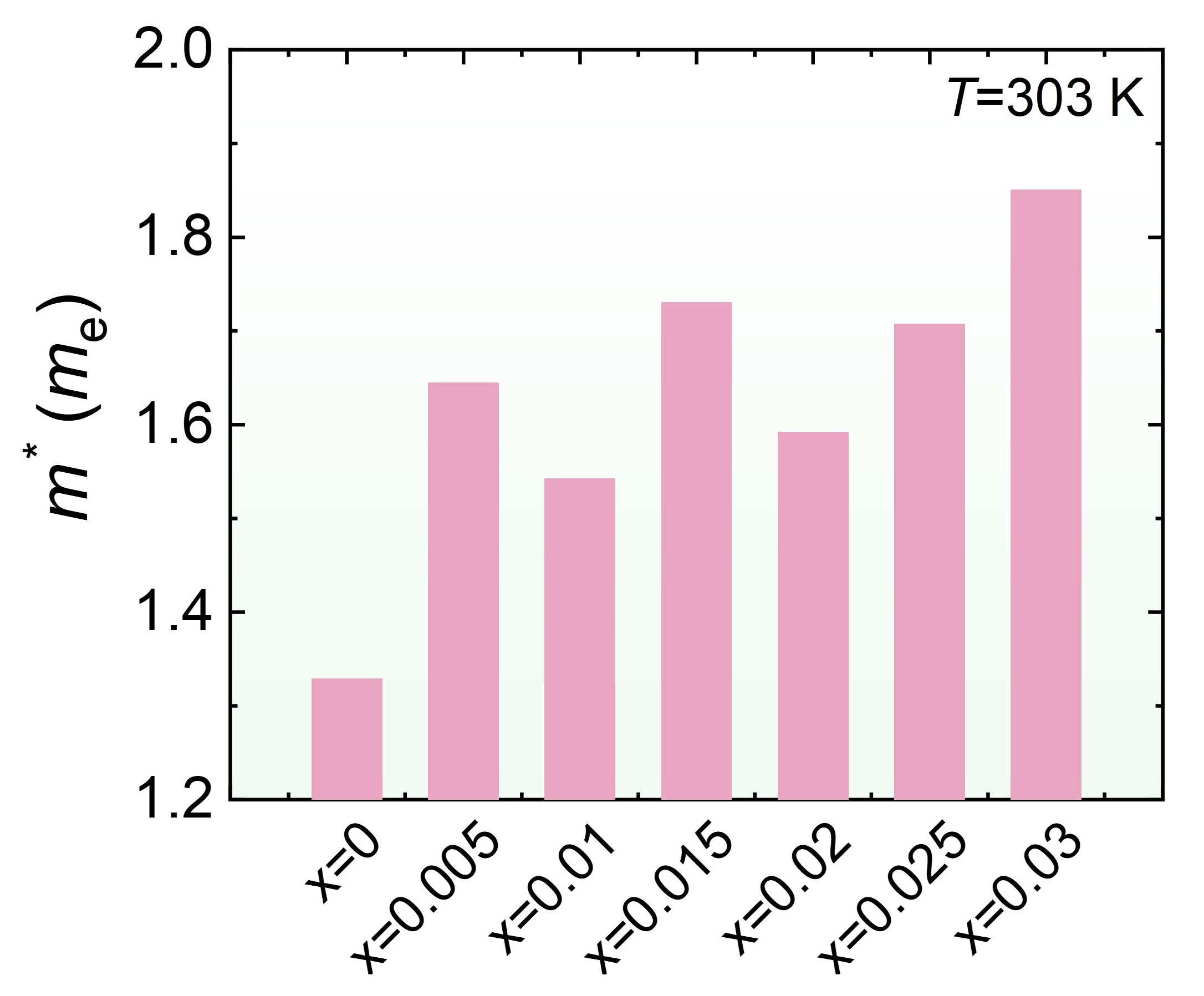


**Figure S7**. Density-of-states (DOS) effective mass (*m*^*^) of Ge_0.94-x_Bi_0.06_Ga_x_Te as a function of Ga content, determined based on the single parabolic band (SPB) model.


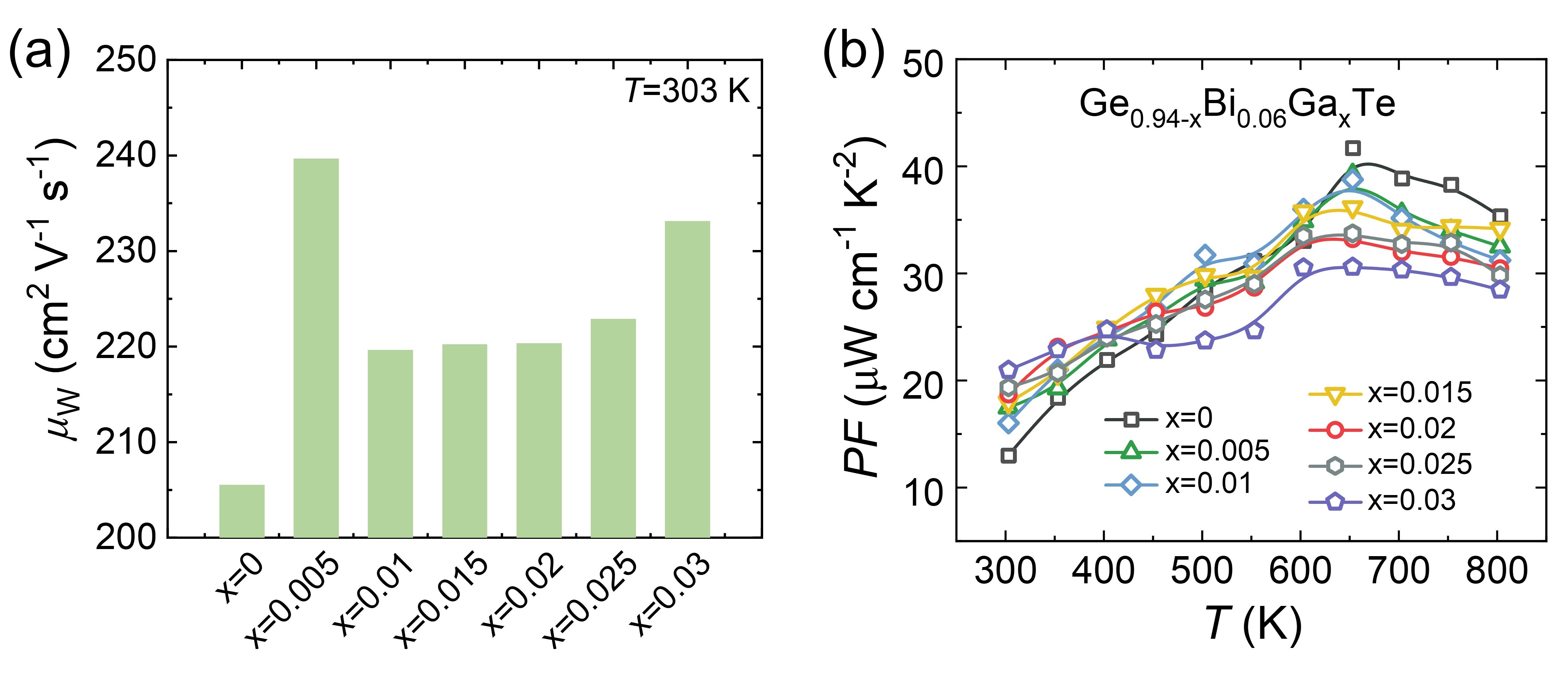


**Figure S8**. (a) Weighted mobility (*μ*_W_) at 303 K and (b) temperature-dependent power factor (*PF*) of Ge_0.94-x_Bi_0.06_Ga_x_Te, highlighting the enhancement of electronic transport with increasing Ga content.

**
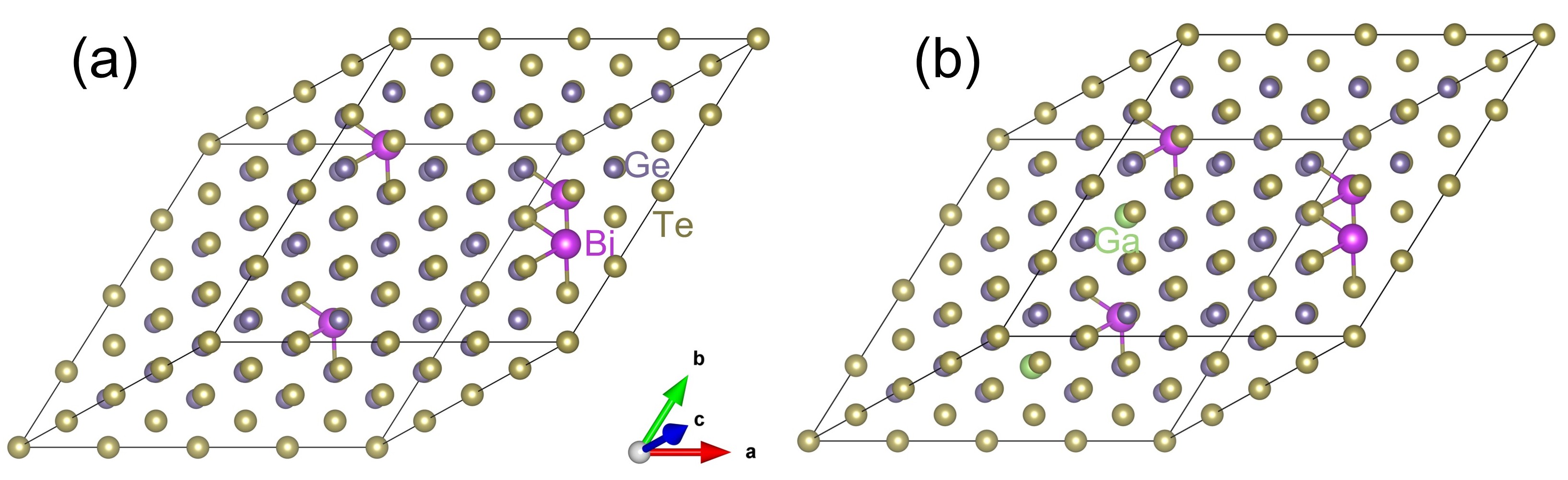
**

**Figure S9**. Supercell models used for DFT calculations: (a) *r-*Ge_60_Bi_4_Te_64_ and (b) *r-*Ge_58_Bi_4_Ga_2_Te_64_.


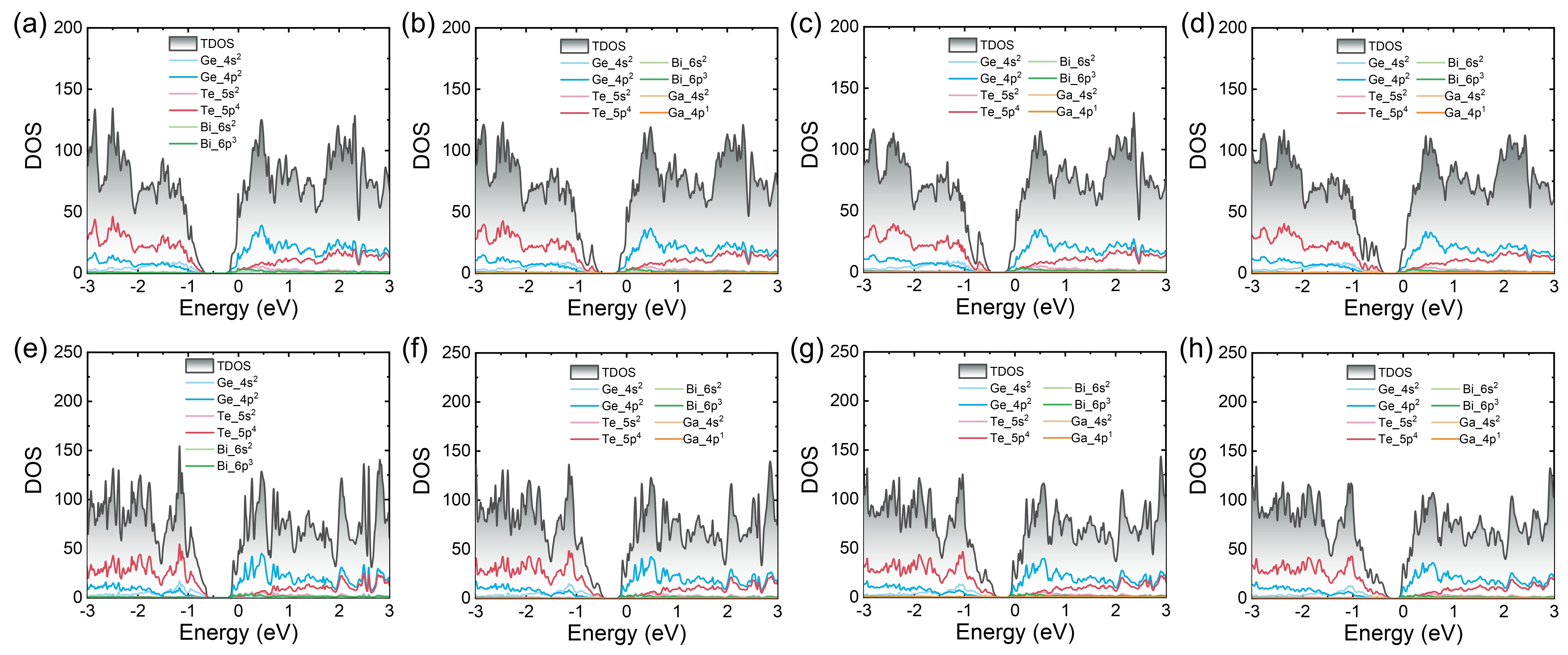


**Figure S10**. Calculated total and projected DOS for rhombohedral structures: (a) *r-*Ge_60_Bi_4_Te_64_, (b) *r-*Ge_59_Bi_4_GaTe_64_, (c) *r-*Ge_58_Bi_4_Ga_2_Te_64_, and (d) *r-*Ge_57_Bi_4_Ga_3_Te_64_; and for cubic structures: (e) *c-*Ge_60_Bi_4_Te_64_, (f) *c-*Ge_59_Bi_4_GaTe_64_, (g) *c-*Ge_58_Bi_4_Ga_2_Te_64_, and (h) *c-*Ge_57_Bi_4_Ga_3_Te_64_.


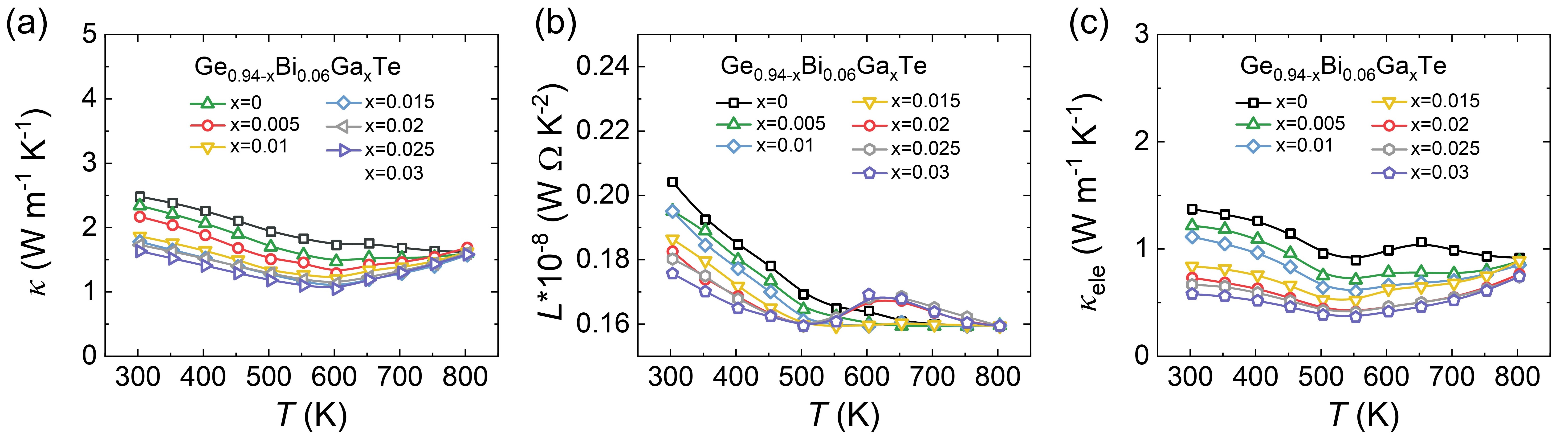


**Figure S11.** Temperature-dependent thermal transport properties of Ge_0.94-x_Bi_0.06_Ga_x_Te: (a) total thermal conductivity (*κ*), (b) Lorenz number (*L*), and (c) electronic thermal conductivity (*κ*_ele_).


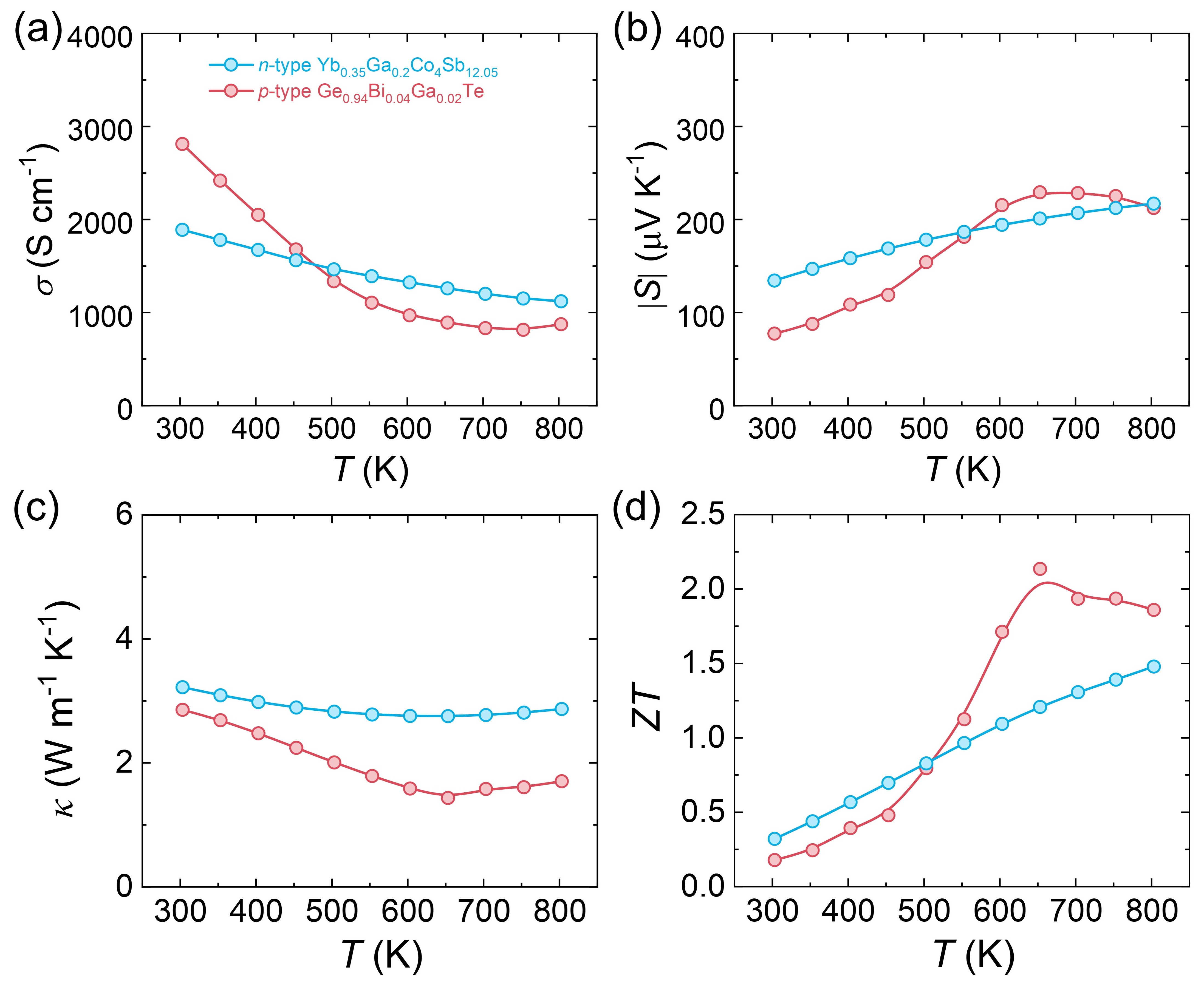


**Figure S12.** Temperature-dependent thermoelectric properties of *n*-type Yb_0.35_Ga_0.2_Co_4_Sb_12.05_ and *p*-type Ge_0.94_Bi_0.04_Ga_0.02_Te: (a) electrical conductivity (*σ*), (b) Seebeck coefficient (*S*), (c) thermal conductivity (*κ*), and (d) *ZT*.

**Table S1.** The comparison of the Ge_0.94_Bi_0.06_Te and Ge_0.92_Bi_0.06_Ga_0.02_Te samples in the longitudinal sound speed (*v*_L_), transverse sound speed (*v*_T_), average sound speed (*v*), and mean free path of phonons (*l*_ph_).

| Samples | *v*_L_ (m s^-1^) | *v*_T_ (m s^-1^) | *v* (m s^-1^) | *l*_ph_ (nm) |  |
| --- | --- | --- | --- | --- | --- |
| Ge_0.94_Bi_0.06_Te | 3633.35 | 1954.25 | 2181.88 | 0.67 | |
| Ge_0.92_Bi_0.06_Ga_0.02_Te | 3475 | 1950.88 | 2170.98 | 0.62 | |

**References**

1 Qin, B., Wang, D., He, W., *et al.*, “Realizing High Thermoelectric Performance in p-Type SnSe through Crystal Structure Modification,” *Journal of the American Chemical Society* 141 no. 2 (2019): 1141–1149, https://doi.org/10.1021/jacs.8b12450.

2 Zhang, F., He, S., Li, R., *et al.*, “Advancing thermoelectrics by vacancy engineering and band manipulation in Sb-doped SnTe–CdTe alloys,” *Applied Physics Letters* 119 no. 17 (2021): 172101, https://doi.org/10.1063/5.0070581.

3 Kresse, G., Furthmüller, J., “Efficient iterative schemes for *ab initio* total-energy calculations using a plane-wave basis set,” *Physical Review B* 54 no. 16 (1996): 11169–11186, https://doi.org/10.1103/PhysRevB.54.11169.

4 Furthmuller, J., Hafner, J., Kresse, G., “Dimer reconstruction and electronic surface states on clean and hydrogenated diamond „100… surfaces,” *Physical Review B* 53 no. 11 (1996): 7334–7351, https://doi.org/10.1103/PhysRevB.53.7334.

5 Kresse, G., Furthmüller, J., “Efficiency of ab-initio total energy calculations for metals and semiconductors using a plane-wave basis set,” *Computational Materials Science* 6 no. 1 (1996): 15–50, https://doi.org/10.1016/0927-0256(96)00008-0.

6 Wang, V., Xu, N., Liu, J.-C., Tang, G., Geng, W.-T., “VASPKIT: A user-friendly interface facilitating high-throughput computing and analysis using VASP code,” *Computer Physics Communications* 267 (2021): 108033, https://doi.org/10.1016/j.cpc.2021.108033.

7 Perdew, J. P., Burke, K., Ernzerhof, M., “Generalized Gradient Approximation Made Simple,” *Physical Review Letters* 77 no. 18 (1996): 3865–3868, https://doi.org/10.1103/PhysRevLett.77.3865.

8 Medeiros, P. V. C., Tsirkin, S. S., Stafström, S., Björk, J., “Unfolding spinor wave functions and expectation values of general operators: Introducing the unfolding-density operator,” *Physical Review B* 91 no. 4 (2015): 041116, https://doi.org/10.1103/PhysRevB.91.041116.

9 Medeiros, P. V. C., Stafström, S., Björk, J., “Effects of extrinsic and intrinsic perturbations on the electronic structure of graphene: Retaining an effective primitive cell band structure by band unfolding,” *Physical Review B* 89 no. 4 (2014): 041407, https://doi.org/10.1103/PhysRevB.89.041407.

10 Bu, Z., Zhang, X., Hu, Y., *et al.*, “An over 10% module efficiency obtained using non-Bi_2_Te_3_ thermoelectric materials for recovering heat of <600 K,” *Energy & Environmental Science* 14 no. 12 (2021): 6506–6513, https://doi.org/10.1039/D1EE02253A.

11 Zhu, J., Zhang, F., Tai, Y., *et al.*, “Enhanced thermoelectric performance and mechanical strength in GeTe enable power generation and cooling,” *InfoMat* 6 no. 4 (2024): e12514, https://doi.org/10.1002/inf2.12514.
